# Evaluation of anti-bacterial effects of nickel nanoparticles on biofilm production by *Staphylococcus epidermidis*

**Published:** 2017-06

**Authors:** Morteza Vahedi, Nima Hosseini-Jazani, Saber Yousefi, Maryam Ghahremani

**Affiliations:** 1 Department of Basic Sciences, Islamic Azad University, Urmia Branch, Urmia, Iran; 2 Department of Microbiology, Faculty of Medicine, Urmia University of Medical Sciences, Urmia, Iran

**Keywords:** *Staphylococcus epidermidis*, Nickel nanoparticles, Biofilm

## Abstract

**Background and Objectives:**

*Staphylococcus epidermidis* produces biofilm by extracellular polysaccharides, causing bacterial adherence to different surfaces. Anti-microbial effects of nickel nanoparticles on some bacterial strains such as *S. aureus* and *Escherichia coli* have been determined in limited studies. The aim of the present study is to examine the inhibitory effect of nickel nanoparticles on biofilm formation using clinical isolates of *S. epidermidis* and its hemolytic effect on human red blood cells.

**Materials and Methods:**

Twenty two *S. epidermidis* isolates were collected and identified by standard microbiological methods. Microtiter plate method was used to determine the biofilm production in bacterial isolates. The amounts of biofilm formation by isolates in the presence of 0.01, 0.05, 0.1, and 1 mg/mL concentrations of nickel nanoparticles were measured. Hemolytic activity of different concentrations of nickel nanoparticles was measured on human RBC suspensions.

**Results:**

Twenty isolates were strong, and two isolates were moderate biofilm producers. Biofilm formation significantly decreased in the presence of 0.05, 0.1, and 1 mg/mL of nickel nanoparticles (p<0.05). Although in the presence of 0.01 mg/mL of nickel nanoparticles, decrease in biofilm formation was observed but it was not statistically significant (p=0.448). Slight hemolytic activity was seen in the presence of nickel nanoparticles.

**Conclusion:**

In this study, the ability of biofilm production was demonstrated for all clinical isolates of *S. epidermidis*. On the other hand, the lowering effects of nickel nanoparticles on biofilm formation were observed.

## INTRODUCTION

*Staphylococcus epidermidis* is a Gram-positive coccus and a member of group of coagulase-negative staphylococci. It is a commensal bacterium that colonizes on the mucous membranes and skin of the human individuals as well as other mammals. *S. epidermidis* is the most prevalent species of staphylococci genus, found in human. At the same time, there are 10 to 24 different strains of *S. epidermidis* at the surface of healthy human body (1), so it is not surprising that *S. epidermidis* is one of the most common contaminating agents in clinical samples. As a commensal bacterium, it has low pathogenic potential. *S. epidermidis* is an opportunistic pathogen which can cause infections only in patients with predisposing factors. It is the major cause of medical implant device infections including intravenous catheters, cerebrospinal fluid shunts, intra-cardiac devices, prosthetic joints, vascular grafts, artificial heart valves, and surgical site infections. On the other hand, this microorganism is one of the causes of keratitis and endophthalmitis, bacteremia, mediastinitis, and other types of infections. *S. epidermidis* is constantly present, with high numbers, on human body surface. Moreover, several virulence factors such as the ability of biofilm production, polysaccharide intercellular adhesion (PIA), biofilm-associated protein, poly-γ-glutamic acid (PGA), staphylococcal enterotoxin-like toxin L (SEIL) and C3 enterotoxin (SEC3), phenol-soluble modulins (PSMs), Clpxp, and extracellular matrix-binding protein can be considered as the main factors which contribute to the development of such infections (2, 3).

Bacterial biofilms are multi-cellular, surface-attached gatherings of bacteria with particular physiologic and architecture characteristics, which cause biofilm resistance to different classes of antibiotics such as penicillins, aminoglycosides, quinolones, and several mechanisms of host defenses. Bacteria in biofilms can resist antibiotics at concentrations, 1000 times higher than those active on the same bacteria in the planktonic state (4). Biofilm formation begins with initial adhesion and further compression into multilayer structures. Thus, formation of biofilms needs adhesion factors for initial colonization of the bacteria on the surfaces and the interaction of cells within this multilayer structure (5). It was estimated that more than 65% of all human infections are biofilm-related (6, 7). Therefore, anti-biofilm agents targeting the biofilm formation are needed.

Nanotechnology is a promising field for production of new types of nanomaterials with medical applications (8). Nanoparticles (NPs) are clusters of molecules, ions, or atoms with diameters in the range of 1–100 nm (9). This size range is so interesting, which gives the opportunity to the particle to penetrate the gap between small molecules and bulk materials. NPs are formed through most periodic table elements. Metallic elements form a large group of NPs (10). Metal nanoparticles (MNPs) have properties that make them suitable for use in medical applications. They are considered as efficient anti-bacterial agents. Many MNPs show anti-bacterial, anti-viral, anti-angiogenesis, and anti-cancer properties, some of which are useful in treating arthritis as well as AIDS. Nanomaterials have a high surface-to-volume ratio which increases their interaction with bacteria and improves their anti-microbial activity (11, 12). Also, anti-biofilm properties of some MNPs have been previously reported (13, 17). Some metals such as gold and silver have been extensively used for MNPs synthesis; however, these elements are so expensive. Nickel (Ni) is cheaper in comparison, but limited studies have demonstrated the anti-bacterial activities of Ni-NPs (18). However, the anti-biofilm effects of Ni-NPs have not been investigated before. In spite of the extensive use of nanoparticles, the probable risks associated with their *in vivo* use have not been investigated completely (19). So, the present study aimed to evaluate the anti-biofilm effects of Ni-NPs on clinical isolates of *S. epidermidis*. Moreover, to determine the adverse effects of Ni-NPs for systemic use in living organisms, the corresponding hemolytic effect was investigated on human red blood cells.

## MATERIALS AND METHODS

### Bacterial isolates.

Twenty two *S. epidermidis* isolates were recovered from clinical specimens submitted to the diagnostic laboratory of Imam Khomeini Educational Hospital of Urmia, Iran, during a six-month period from November 2014 to July 2015. *S. epidermidis* isolates were identified based on bacterial morphology after Gram staining via light microscopy, appearance of colonies on selective media, non-haemolytic colonies on blood agar, positive catalase and urease, weak nitrate reduction, negative coagulase and oxidase, and sensitivity to novobiocin (20).

### Nickel Nanoparticles.

Ni-NPs were purchased from Sigma-Aldrich, in the form of nanopowder, suspended in TSB (Merck) and ultrasonicated for 2 h before use. The average size of particles was less than 100 nm.

### Determination of antibacterial activity of Ni-NPs.

MIC and MBC against each isolate were determined by preparing serial dilutions using Mueller Hinton Broth (BBL). The concentration range for determining MIC and MBC values for Ni-NPs was considered 2–0.008 mg/mL. Bacterial inoculates were added to serial dilutions of Ni-NPs (Sigma-Aldrich), with final concentration of 1.5×10^6^ CFU/mL. Experiments were carried out in triplicate.

### Microtiter dish biofilm formation assay.

Bio-film formation assay was performed by the method proposed by O’Toole with some modifications (21). In brief, the isolates were grown in TSB for 24 h at 37°C, and then the cultures were diluted 1:100 in fresh medium for biofilm assays. 200 μL of the dilution was added to each well in a U-bottom shape 96-well dish. For each isolate, eight replicates were used. The microtiter plates were incubated overnight at 37°C. After incubation, planktonic cells were removed by tapping down the plate and removing the residual liquid; in the next step, the plates were submerged into a small tub of water, and the water was removed. The process was replicated for three times. 200 μL of crystal violet solution in water (0.1%) was added to each well, and the plates were incubated at room temperature for 15 min, then rinsed submerging in a water tub 3–4 times, shacked out, and then tapped down on paper towels in order to remove all unattached materials. Microtiter plates were turned upside down and dried overnight. In order to quantify the biofilm amounts, 250 μL of acetic acid solution (30% in water) was added to each well to solve the dye, and the plates were incubated at room temperature for 10 min. Well’s contents were transferred into a new flat bottom microtiter dish. Absorbance was measured by ELISA reader (Awareness Technology Inc) at 550 nm. Acetic acid solution (30% in water) was used as the blank. *S. epidermidis* ATCC 14990 was used as the biofilm producer strain. The plate containing culture medium was used as negative control and the absorbance of crystal violet binding to the wells was measured.

Interpretation of biofilm production was based on criteria explained by Stepanovic et al. (22). Briefly, the optical density cut-off (ODc) value is considered as average OD of negative control + 3 × SD (standard deviation) of negative control, and the biofilm producers were classified as presented in Table 1. ODs were the average of the optical density of eight replicate samples.

**Table 1.** Criteria used for the classification of isolates in respect to their biofilm formation

**Criteria**	**Biofilm-formation capacity**
ODs ≤ODc	No biofilm producer
ODc≤ ODs ≤ 2×ODc	Weak biofilm producer
2 × ODc ≤ ODs ≤ 4 × ODc	Moderate biofilm producer
ODs> ODc×4	Strong biofilm producer

### Susceptibility of biofilm to nickel nanoparticles.

Biofilm was prepared in microtiter plates, as described above. Different concentrations of Ni-NPs (0.01, 0.05, 0.1, and 1 mg/mL) were added to different wells for the same isolate. The same protocol was used for quantification of biofilm amounts (21, 23). For each treatment, eight replicates were used.

### Hemolytic activity of nickel nanoparticles.

Hemolytic activity of Ni-NPs was measured using the method described before (24). In brief, final concentrations of 0.01, 0.05, 0.1 and 1 mg/mL of Ni–NPs were separately prepared by addition of 0.8 mL of NPs to 0.2 mL of diluted human RBC suspensions. In order to prepare the negative and positive control groups, 0.8 mL of PBS and distilled water were added to 0.2 mL of diluted RBC suspensions, respectively. The samples were gently vortexed and incubated at 37±0.5°C for 150 min, and then centrifuged at 3500 rpm for 5 min. The absorbance of hemoglobin was measured at 577 nm after transferring 100 μL of supernatant from each tube to a 96-well microplate. The percent of erythrocytes hemolysis was calculated using the following formula. All the experiments were performed in triplicate.

percent hemolysis (%)=[(sample absorbance−negative control)/(positive control−negative control)]×100%

### Statistical analysis.

A comparison was made between the mean of optical density obtained for each isolate in the presence or absence of Ni-NPs. The data were statistically analyzed by SPSS (16). Oneway analysis of variance (ANOVA) was used for analyzing the group means. Differences between means were considered statistically significant at p<0.05.

## RESULTS

The sources of investigated isolates with respect to hospital wards, sample type, and gender of patients are provided in Table 2.

**Table 2.** Demographic characteristics related to the source of studied *S. epidermidis* isolates

**Demographic characteristics**		**No. (%)**	
**Gender**	Male	10 (45.5)	
	Female	12 (54.5)	

**Sample type**	Blood	6 (27.3)	
	Urine	15 (68.2)	
	Synovial fluid	1 (4.5)	

**Source of Sample**	Out patients	2 (9)	
	In patients	Emergency	5 (22.8)
		Urology	1 (4.60)
		I.C.U	1 (4.60)
		Internal	2 (9)
		Nephrology	11 (50)

### Determination of anti-bacterial activity of Ni-NPs.

No anti-bacterial effects were detected in the above-mentioned concentrations of Ni-NPs (Table 3). There were not any relations between anti-bacterial and anti-biofilm effects of Ni-NPs in the tested concentrations.

**Table 3.** Anti-microbial activity and inhibition of biofilm formation by different concentrations of Ni-NPs on the studied isolates and *S. epidermidis* ATCC14990

**No. of isolates**	**Biofilm-formation Capacity**	**MIC/MBC of Ni-NPs (mg/mL)**	**OD550±SD Microtiter Dish Biofilm Formation Assay (in the absence of Ni-NPs)**	**OD550±SD Microtiter Dish Biofilm Formation Assay (in the presence of 0.01 mg/mL Ni-NPs)**	**OD550±SD Microtiter Dish Biofilm Formation Assay (in the presence of 0. 05 mg/mL Ni-NPs)**	**OD550±SD Microtiter Dish Biofilm Formation Assay (in the presence of 0.1 mg/mL Ni-NPs)**	**OD550±SD Microtiter Dish Biofilm Formation Assay (in the presence of 1 mg/mL Ni-NPs)**
1	Strong biofim Producer	NE^*^	0.34±0.0159	0.32±0.0025	0.15±0.0186	0.13±0.0223	0.13±0.0124
2	Strong biofim Producer	NE	0.12±0.0152	0.13±0.0015	0.07±0.0121	0.06±0.0163	0.07±0.0140
3	Strong biofim Producer	NE	0.17±0.0345	0.12±0.0030	0.05±0.0320	0.08±0.0182	0.08±0.0123
4	Moderate biofilm Producer	NE	0.06±0.0176	0.05±0.0157	0.03±0.0059	0.03±0.0079	0.05±0.0148
5	Strong biofim Producer	NE	0.17±0.0532	0.14±0.0135	0.06±0.0284	0.05±0.0167	0.2±0.0147
6	Strong biofim Producer	NE	0.36±0.0231	0.27±0.0133	0.02±0.0028	0.02±0.0067	0.02±0.0290
7	Strong biofim Producer	NE	0.35±0.0844	0. 3±0.0043	0.17±0.0368	0.15±0.0088	0.25±0.0131
8	Moderate biofilm Producer	NE	0.26±0.0087	0.18±0.0146	0.15±0.0294	0.12±0.0066	0.22±0.0049
9	Strong biofim Producer	NE	0.31±0.0410	0.17±0.0167	0.12±0.0092	0.1±0.0222	0.25±0.0148
10	Strong biofim Producer	NE	0.30±0.0370	0.21±0.0183	0.09±0.0255	0.07±0.0620	0.22±0.0278
11	Strong biofim Producer	NE	0.22±0.0181	0.21±0.0147	0.20±0.0453	0.18±0.0440	0.17±0.0431
12	Strong biofim Producer	NE	0.23±0.0370	0.20±0.0184	0.20±0.0482	0.18±0.0427	0.18±0.0329
13	Strong biofim Producer	NE	0.29±0.0094	0.22±0.0207	0.16±0.0275	0.14±0.0325	0.14±0.0196
14	Strong biofim Producer	NE	0.37±0.0584	0.3±0.0256	0.16±0.0179	0.15±0.0164	0.15±0.0227
15	Strong biofim Producer	NE	0.22±0.0960	0.2±0.0098	0.16±0.0391	0.21±0.0271	0.23±0.0416
16	Strong biofim Producer	NE	0.19±0.0281	0.18±0.0148	0.18±0.0826	0.14±0.0068	0.14±0.0442
17	Strong biofim Producer	NE	0.34±0.0248	0.32±0.0209	0.20±0.0210	0.19±0.0159	0.18±0.0212
18	Strong biofim Producer	NE	0.30±0.0142	0.26±0.0166	0.23±0.0989	0.21±0.0062	0.21±0.0601
19	Strong biofim Producer	NE	0.24±0.0400	0.23±0.0199	0.16±0.0200	0.12±0.0092	0.19±0.0377
20	Strong biofim Producer	NE	0.21±0.0335	0.19±0.0154	0.16±0.0190	0.13±0.0375	0.15±0.0219
21	Strong biofim Producer	NE	0.27±0.0565	0.26±0.1191	0.19±0.0518	0.17±0.0160	0.16±0.0261
22	Strong biofim Producer	NE	0.5±0.0332	0.15±0.0114	0.10±0.0299	0.09±0.0092	0.1±0.0404
*S. epidermidis* ATCC14990	Strong biofim Producer	NE	0.31±0.0076	0.26±0.0601	0.12±0.0457	0.12±0.0063	0.12±0.0052
Total		NE	0.267±0.0342	0.212±0.0205	0.136±0.0503	0.123±0.0198	0.157±0.0249

NE^*^: No antibacterial effect was seen in studied concentrations of Ni-NPs.

### Determination of biofilm-producing isolates.

A total number of 22 *S. epidermidis* isolates were investigated with respect to their ability in biofilm formation. Twenty isolates were strong biofilm producers, and two isolates were moderate (Table 3). *S. epidermidis* ATCC14990 was a strong biofilm producer. So, all isolates were studied to determine the effect of nickel nanoparticles on biofilm production. There were no significant differences in the amounts of biofilm formation between the isolates regarding different wards or clinical samples (p>0.05).

### Effects of Ni-NPs on bacterial biofilm growth.

**Fig. 1.**
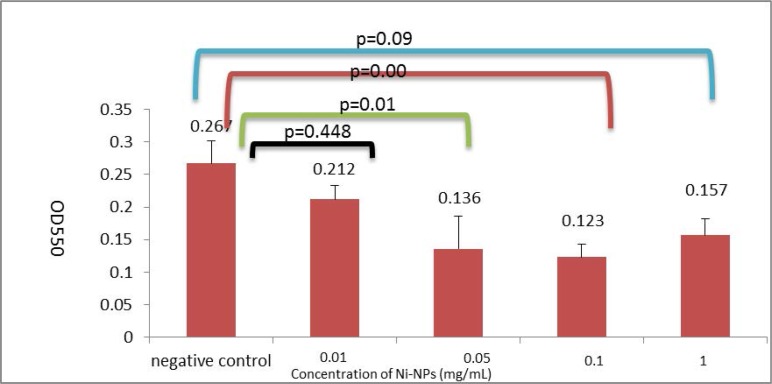
Biofilm formation in the presence or absence of Ni-NPs

Fig. 1. shows the mean optical density±SD for each group in the presence of 0, 0.01, 0.05, 0.1, and 1 mg per mL of Ni-NPs. According to the statistical analysis, the effects of 1, 0.1 and 0.05 mg per mL of Ni-NPs on biofilm reduction were significant (P<0.05), but the effect of 0.01 mg/mL of Ni-NPs on biofilm reduction was not significantly different compared with the non-treated group (p=0.448). There were not significant differences in biofilm production between groups treated with 0.05, 0.1 and 1 mg per mL of Ni-NPs (p>0.05).

### Hemolytic activity of nickel nanoparticles.

**Fig. 2.**
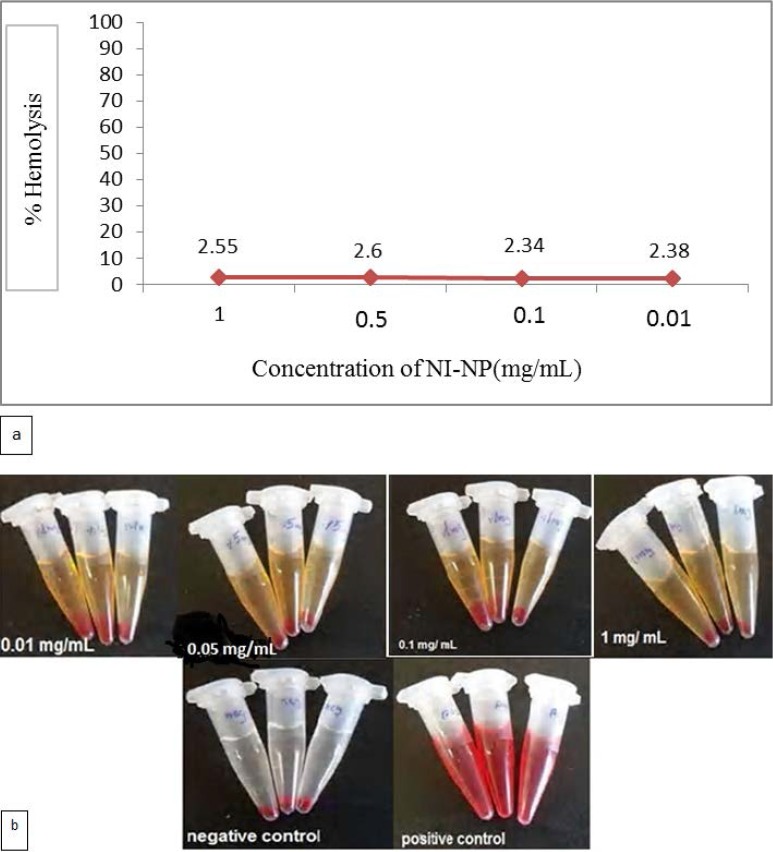
The percentage hemolysis of human erythrocytes (a), Photographs of human erythrocytes were incubated with Ni-NPs with different concentrations (0.01, 0.05, 0.1 and 1 mg/mL) with 150 min exposure time (b).

The hemolytic activity of 0.01, 0.05, 0.1 and 1 mg/mL of nickel nanoparticles on human RBC was 2.38%, 2.34%, 2.6% and 2.55%, respectively (Fig. 2).

## DISCUSSION

Nickel is usually used in the preparation of stainless steel and other nickel alloys, so its properties in pure form are not well known. Alloys containing nickel show more resistance to both heat and corrosion and are used in chemical industry, food processing (storage tanks, piping, etc.), and medical devices (pacemakers, orthopedic implants, needles, and surgical instruments) (25, 27).

Different types of nanoparticles such as copper, zinc, titanium, silver, and nickel have proven to be acticve against bacteria, viruses and other eukaryotic microorganisms (28, 35); some are effective in eradication of bacterial biofilms as well (14, 36, 37). In recent years, different ways have been developed for eradication of biofilms. NPs are useful approaches for controlling biofilms. Some NPs, for example silver NPs, elicit bactericidal effects as well as remarkable therapeutic efficacy in killing biofilm-producing bacteria (23, 38). The anti-bacterial effects of Ni-NPs have been shown elsewhere (18); however, the anti-biofilm properties of Ni-NPs have not been investigated so far. *S. epidermidis* is one of the common causes of hospital-acquired infections, especially in patients with catheters or other surgical implants (39). The capacity of *S. epidermidis* to produce biofilm is an important factor in infectivity. Considering the ability of *S. epidermidis* to form biofilms on viable and non-viable surfaces, especially on plastic devices as its major virulence factor (40), the present study was designed to determine the inhibiting effects of Ni-NPs on biofilm growth of clinical isolates of *S. epidermidis.*

In 2009, Perrin et al. reported that sub-inhibitory concentration of nickel induces *E. coli* cells to change their lifestyle, producing biofilms rather than growing as planktonic cells on polystyrene and stainless steel coupons (41). However, the properties of NPs could be completely different compared to microparticles or bulk because, NPs have a very large surface area and high particle number per unit mass (42).

Argueta-Figueroa et al. prepared nickel (Ni) and bimetallic Cu–Ni NPs by a simple chemical method and investigated their anti-bacterial activity against *S. aureus*, *S. mutans*, and *E. coli.* They found similar anti-bacterial behavior for Ni and Cu–Ni NPs. They could not achieve a complete inhibition of bacterial growth (MBC) even at the highest tested concentration of NPs (1000 μg/mL), but with both types of NPs, the MIC for tested bacterial strains was 1000 μg/mL. They concluded that NPs are active against tested strains and proposed their potential use in dental materials (18). The current findings partly confirmed the findings of other researchers, showing that even the highest studied concentrations of Ni-NPs did not inhibit the complete growth (MBC) of tested isolates or did not inhibit even making the broth medium turbid (MIC) (Table 3). However, the anti-biofilm properties of Ni-NPs or nickel nanoalloys have not been investigated before.

Different NPs with anti-bacterial effects probably use different mechanisms for their anti-bacterial action. Silver NPs are broad spectrum anti-bacterial agents. They can cause destabilization of the outer membrane of bacteria and reduce the levels of adenosine triphosphate (ATP) in cells under exposure (43, 44). However, the specific anti-bacterial mechanism of Ni-NPs has not been illustrated yet.

In this study, the safety of Ni-NPs was investigated on a limited basis by measuring the hemolytic activity of Ni-NPs. Only negligible hemolytic effects were seen in the tested concentrations (Fig. 2). However, considering reports on the toxic effects of nickel as a risk factor for cancers of lung and sinus, especially after occupational exposure to nickel via inhalation (42) and lack of extensive studies on safety of nickel nanoparticles usage in human or animal model, complementary studies are recommended to address the use of Ni-NPs on the surfaces of catheters, the prominent surfaces for colonization of resistant isolates of *S. epidermidis.*

The present study is the first investigation on the inhibitory effects of Ni-NPs on biofilm production. The results indicated the ability of *S. epidermidis* clinical isolates in biofilm production. Also, the effect of suitable Ni-NPs concentrations in reduction of biofilm was demonstrated.
